# The structural pathways from AI attitude to AI literacy among university students: considering the serial mediators of AI self-efficacy and AI motivation

**DOI:** 10.3389/fpsyg.2026.1955267

**Published:** 2026-10-08

**Authors:** Xu Wang, Jinpeng Niu

**Affiliations:** 1 School of Humanities, Shandong Institute of Petroleum and Chemical Technology, Dongying, Shandong, China; 2 Faculty of Education, Southwest University, Chongqing, China

**Keywords:** AI attitude, AI literacy, AI motivation, AI self-efficacy, serial mediation

## Abstract

**Background:**

In the digital era, university students' artificial intelligence (AI) literacy has increasingly received considerable scholarly attention. However, the existing literature predominantly focused on extrinsic supportive conditions that influence AI literacy development, and very rare research has examined how subjective AI attitudes relate to AI literacy acquisition. In view of this, the current study aims to explore the structural relations between AI attitude, AI self-efficacy, AI motivation, and AI literacy among contemporary university students.

**Methods:**

A chain mediation model was employed as the statistical technique, and the participants were 1,323 college students selected from two universities in Shandong, China.

**Results:**

The results demonstrated that AI attitude is positively associated with AI literacy both directly and indirectly through the single mediation of AI self-efficacy, the single mediation of AI motivation, and the serial mediations of AI self-efficacy and AI motivation. Nevertheless, due to the limitations of unrecorded classroom-level clustering, the statistical findings should be treated as exploratory associations.

**Conclusion:**

The research underscores the importance of addressing AI-related psychological factors, rather than solely focusing on external supports, in order to effectively promote students' AI literacy in higher education system. Additionally, this exploratory study suggests that the preliminary associations among AI attitude, AI self-efficacy, AI motivation, and AI literacy may require more rigorous replication using hierarchically structured data with recorded cluster identifiers in the future.

## Introduction

In the context of digital technology reshaping the global educational landscapes, artificial intelligence (AI) has transformed from a merely supplementary tool into a core medium within the higher education ecosystem, profoundly influencing the cognitive development and learning paradigms of college students (Bewersdorff et al., 2025a; Jia and Tu, 2024; Kong et al., 2023). With the widespread application of AI tools such as Chat Generative Pre-Trained Transformer (ChatGPT) and Claude, the ability to critically navigate, evaluate, and utilize AI, widely recognized as AI literacy, has become a core competency for university students to maintain academic achievement and professional competitiveness in the digital era (Kong et al., 2023; Long and Magerko, 2020). Although the proliferation of AI technologies yields efficiency dividends, a lack of essential AI literacy among university students may not only increase the risk of academic integrity violations and the weakening of critical thinking (Deep and Chen, 2025), but can also make them more vulnerable to structural unemployment in a highly automated future labor market (Wut et al., 2025). Given this context, exploring the internal psychological mechanism of college students' AI literacy holds significant practical implications for enhancing the adaptability of global higher education systems.

Prior research has primarily highlighted the driving role of macro-level external interventions on AI literacy, emphasizing factors like family regulation, classroom climate, and instructional practices (Shen and Cui, 2024; Wang X. et al., 2025; Zhang et al., 2026). However, the efficacy of these external interventions ultimately depends on the individual's internal micro-level psychological processes. This endogenous mechanism cannot be overlooked in competency construction (Zimmerman, 2000). Yet, research exploring the formation mechanisms of AI literacy from an internal micro-perspective remains relatively limited. For instance, a few studies have demonstrated that AI attitude, defined as an individual's perceived and emotional response to AI technology and its applications (Grassini, 2023a), shows a positive correlation with AI literacy levels (Li et al., 2025). Also, AI self-efficacy, an individual's confidence in the ability to complete AI-related tasks (Wang and Chuang, 2024), positively links to AI literacy, specifically manifesting in the mastery of complex technologies and practical application capabilities (Kong et al., 2025). Further, AI motivation is positively associated with AI literacy through individual engagement and persistence in AI-related activities (Li et al., 2026).

Although the academic community has preliminarily explored corresponding associations between AI attitude, AI self-efficacy, AI motivation, and AI literacy, two critical gaps remain in the current research field. Firstly, the existing literature is largely confined to fragmented analyses of single variables or surface-level examinations of bivariate relationships. Researchers have often focused solely on the direct role of AI attitude on AI literacy (Li et al., 2025) or explored the mediating role of AI self-efficacy in isolation (Asio, 2025). Such fragmented approaches fail to systematically reveal the chain mediations, spanning from attitude assessment to self-efficacy construction, motivation arousal, and literacy enhancement. Understanding such a holistic sequence is crucial because it more accurately and ecologically reflects the complete process of how internal psychological dynamics relate to adaptive behavioral capacities. Secondly, most existing literature relies on traditional technology acceptance models (TAM), overly focusing on how the physical hardware attributes and object functions of AI technology itself drive user behavior (Chen et al., 2020; Kamruzzaman et al., 2023; Ouyang et al., 2022), while largely neglecting the complex development of the internal pathways. The deep interaction between college students and AI is not merely the adoption of a tool, but a profound psychological shift accompanied by cognitive restructuring and emotional adaptation. Facing highly autonomous intelligent systems (Bai and Wang, 2025), it remains unclear how college students' AI attitudes bridge the surface layer of technology acceptance to associate with internal AI motivation through the mediating role of AI self-efficacy. This intrinsic mechanism concerning the iterative upgrade of cognitive frameworks has yet to undergo systematic empirical testing. Merely exploring the physical aspects of external technological attributes is clearly insufficient to comprehensively explain the differences in core competencies exhibited by college students in complex human-machine collaborative environments. To address these research limitations, the present study aims to construct a sequential multiple mediation model. This model systematically examines how AI attitude relates to AI literacy through the serial mediating roles of AI self-efficacy and AI motivation.

## Theoretical foundation

This study integrates Bandura's self-efficacy theory and Katz et al.'s uses and gratifications theory (UGT) to construct a comprehensive theoretical model explaining the pathways of college students' AI literacy. Self-efficacy serves as the core mechanism moderating the relationship between personal factors which encompass individuals' cognitive tendencies and psychological attributes and their ultimate behavioral performance, directly determining the level of effort and persistence that individuals demonstrate when confronting challenges (Bandura, 1997). When individuals encounter AI technology, their evaluative attitudes constitute typical personal factors, and this psychological inclination directly shapes how they process technological information. A positive AI attitude can elicit favorable cognitive appraisals and alleviate negative emotional arousal during technology exposure, thereby enhancing an individual's confidence in their ability to complete specific AI tasks. This state of confidence is referred to as AI self-efficacy (Wang and Chuang, 2024; Zimmerman, 2000). Empirical evidence further confirms that a receptive appraisal of emerging technologies is a prerequisite for individuals to build operational confidence and technological competence (Ng et al., 2024). Therefore, the cognitive translation from evaluative attitudes to efficacy beliefs accurately reveals how psychological readiness lays the foundation for the subsequent engagement and competency development.

The UGT further elucidates the underlying mechanisms through which cognitive efficacy expectations translate into diverse behavioral driving forces. Originating from communication psychology and media adoption paradigms, UGT posits that individuals are active media agents who purposefully select and utilize technologies to satisfy specific utilitarian, social, diversionary, and psychological needs (Katz et al., 1973). In digital environments, users do not interact with emerging tools passively; rather, their technology utilization is propelled by an array of distinct motives encompassing instrumental problem-solving, cognitive diversion, interpersonal interaction, and affective management (Sundar and Limperos, 2013). When individuals possess high AI self-efficacy, their confidence in mastering technological challenges activates proactive expectations of gratification, thereby stimulating their general motivation to employ AI across varied contextual demands. This efficacy belief functions as a fundamental cognitive antecedent that awakens learners' intention to harness AI for pragmatic tasks, entertainment, stress alleviation, and social connectivity (Kang et al., 2024). In intelligent learning settings, such a comprehensive motivational orientation provides the continuous behavioral impetus necessary to sustain ongoing technological experimentation. Through this expectation-to-gratification pathway, initially static efficacy evaluations are effectively changed into active purposive drives, achieving a seamless progression from cognitive self-assurance to goal-directed motivational arousal.

Integrating self-efficacy theory and UGT reveals a coherent serial pathway from cognitive appraisal to multidimensional motivational activation, and to the output of higher-order competencies. Self-efficacy theory explains how favorable AI attitude benefits AI self-efficacy, establishing the fundamental belief system necessary for goal-directed action. Concurrently, UGT clarifies how this efficacy perception mobilizes a composite spectrum of user motivations, spanning instrumental utility, experiential enjoyment, social interaction, and tension release. Because developing comprehensive AI literacy requires not only operational mastery but also critical reflection and ethical judgment (Su et al., 2023), sustained cognitive effort and exploration are essential. The broad motivational drive mobilized by self-efficacy sustains students' active experimentation and diverse technology engagements (Ng et al., 2024), providing the experiential foundation needed for complex problem-solving and ethical evaluation (Kong et al., 2023). Through this psychological mechanism, evaluative attitudes are effectively translated through competence beliefs and multifaceted motivations into higher-order literacy outcomes. By systematically integrating cognitive appraisals, efficacy expectations, and functional motivation profiles, this analytical framework provides a rigorous theoretical foundation for explaining how relating AI attitude to AI literacy among university students.

## Literature review

### AI attitude and AI literacy

Amid the wave of intelligent technologies reshaping educational paradigms, the connotation of literacy has expanded from traditional reading and writing abilities to a composite competency structure encompassing algorithmic comprehension and human-machine collaboration (Almatrafi et al., 2024). As a core competency for digital survival, AI literacy goes beyond mere technical operations. It is conceptualized as a comprehensive capability framework enabling individuals to critically evaluate AI technologies, communicate and collaborate efficiently with intelligent systems, and utilize AI to solve problems in complex and dynamic real-world scenarios (Ng et al., 2024). According to foundational research in this field, AI literacy includes an understanding of basic AI principles, ethical reflection on algorithmic decision-making, and the practical ability to apply AI in work and study (Long and Magerko, 2020). Kong et al. (2023) further emphasized the importance of the cognitive dimension of AI literacy, refining it as the capacity to understand AI concepts, evaluate them, and apply them to interpret the real world. Current academic consensus tends to view AI literacy as a dynamically developing cognitive schema. A series of systematic reviews in education summarize it as the comprehensive manifestation of knowledge, skills, methodological processes, emotional tendencies, and values that individuals gradually internalize during AI education (Casal-Otero et al., 2023; Tan and Tang, 2025). Moreover, empirical studies indicate that AI literacy is not an isolated set of skills but a four-dimensional structure involving cognition, emotion, behavior, and ethics. This complex, multidimensional nature dictates that its formation process is profoundly influenced by individual psychological characteristics (Ng et al., 2024).

AI attitude, serving as an individual's evaluative psychological inclination toward AI technology and its social impacts (Grassini, 2023a), is a deep-seated endogenous factor driving the development of AI literacy. From a psychological construction perspective, AI attitude is a complex multidimensional entity composed of cognitive, affective, and behavioral tendencies. It encompasses an individual's identification with the practical value of AI and the psychological responses generated during interaction (Schepman and Rodway, 2023). A series of empirical studies on college students has shown that individuals' AI attitudes are positively associated with their AI literacy levels across both general contexts and specific fields like robotics (Hornberger et al., 2023; Wang et al., 2023). A review by Tang et al. (2023) notes that college students' perceived usefulness and ease-of-use attitudes toward AI technologies are core antecedents predicting their mastery of AI knowledge. When college students hold an open and positive AI attitude, this psychological inclination is often associated with an exploratory aspect, encouraging them to deeply understand the underlying principles and ethics of AI, thereby accumulating a higher level of AI literacy (Chang and Hwang, 2025; Li et al., 2025). Conversely, students with negative or resistant attitudes toward AI often exhibit distinct technology-avoidance tendencies. Grassini (2023b) points out that students' initial perceptions of AI tools strongly influence whether they treat them as innovative learning companions or academic threats. A distrustful attitude can hinder individuals from deeply processing AI knowledge (Park, 2026), subsequently constraining the enhancement of their AI literacy. This aligns with findings from previous reviews indicating a negative correlation between negative attitudes toward AI and the ability to correctly identify and effectively use AI-related products (Ng et al., 2021). Therefore, AI attitude is not only the first psychological threshold for individuals encountering AI but also a crucial factor shaping whether AI literacy can transition from superficial tool usage to deep competency internalization.

### AI self-efficacy as a potential mediator

General self-efficacy is a core driving force in Social Cognitive Theory (SCT), representing people's general beliefs in their skills and capabilities to cope with challenging situations (Bandura, 1997). In addition to general self-efficacy, task-specific self-efficacy measures a personal level of confidence in a particular task or context, such as internet self-efficacy, computer self-efficacy (Torkzadeh and Van Dyke, 2001), and information and communication technology self-efficacy (Musharraf et al., 2018). Building on these technology-related self-efficacy studies, AI self-efficacy is defined as an individual's general belief in their ability to use and interact with AI (Wang and Chuang, 2024). Extensive research in educational psychology shows that higher self-efficacy is positively correlated with motivation, persistence, and academic achievement (Waddington, 2023). This phenomenon is particularly evident in emerging fields like AI, such as in studies exploring how adolescents' AI self-efficacy relates to AI literacy levels (Tian et al., 2026). Research indicates that individuals with high self-efficacy are more likely to actively address new challenges, persist in their efforts, and effectively adapt to technological advancements (Li et al., 2025; Wallin et al., 2021). Li and Kim (2024) further note that in an era of technological uncertainty, individuals with high AI self-efficacy are more inclined to set goals, such as acquiring or cultivating new technical skills like AI literacy. The aforementioned studies are consistent with the findings of a cross-national comparative study, concluding that AI self-efficacy can significantly predict individuals' AI literacy levels (Low et al., 2025).

Learners' attitudes toward AI profoundly influence the construction of AI self-efficacy, generally through pathways of emotional arousal and cognitive appraisal. According to Bandura's theory of self-efficacy regulation (Bandura, 1997), a positive attitude toward technology can reduce an individual's anxiety when facing emerging complex technologies, thereby enhancing their efficacy expectations by improving their emotional state (Scherer et al., 2019). Individuals often hold dual perceptions of AI, where positive expectations regarding technology-enabled efficiency coexist with negative concerns about ethical and occupational risks (Schepman and Rodway, 2020). When students possess extensive AI usage experience and maintain an optimistic stance, this positive attitude can effectively suppress technophobia (Pinto dos Santos et al., 2019), deepening their understanding of AI mechanisms and building their confidence to navigate intelligent systems (Sassis et al., 2021). This positive AI attitude significantly elevates students' self-efficacy when performing complex AI tasks (Atalla et al., 2025), allowing them to demonstrate greater command in human-machine collaboration. In contrast, negative attitudes toward AI often elicit psychological resistance to technology replacing human roles (Atalla et al., 2025; Schepman and Rodway, 2020). This defensive psychology not only diminishes willingness to interact but also contributes to severe deficits in confidence when confronting intelligent tools. Empirical research further confirms that a strong interest in AI, frequent usage experience, and a positive attitude play vital roles in fostering AI self-efficacy (Bewersdorff et al., 2025a). In summary, while a positive AI attitude provides the initial willingness to participate, learners can only cross the conceptual gap from psychological inclination to actual capability realization when this willingness relates to a reserve of confidence for handling specific AI tasks.

### AI motivation as a potential mediator

Individuals' behavioral intentions and performance are profoundly influenced by their internal psychological dynamics. AI motivation is conceptualized not merely as a singular intention to use technology, but rather as a multidimensional construct reflecting the specific gratifications individuals seek from technology, encompassing instrumental, entertainment, social, and escape motives (Katz et al., 1973; Sundar and Limperos, 2013). In human-machine interaction practices, students often exhibit different types of utilization orientations (Scherer and Siddiq, 2019). Some learners are primarily driven by instrumental utility and problem-solving, while others are motivated more by entertainment, social connection, or the need to escape academic stress (Li et al., 2025). This differentiation in motivation types stems fundamentally from variations in how individuals perceive the utility and gratification potential of AI tools to meet their specific contextual demands. Prior research indicates that when interacting with AI systems, the arousal of diverse use motivations is closely linked to the perceived capability to master these technologies (Chiu et al., 2024), underscoring the importance of optimizing AI learning environments to support students' operational confidence (Bergdahl et al., 2020). When learners' self-assessed capabilities enable them to successfully navigate intelligent systems, the resulting goal-directed use motivations can significantly promote the enhancement of digital literacy across multiple dimensions (Chiu et al., 2022). Recent studies further confirm that active engagement driven by distinct utilitarian and recreational motives has a substantial positive impact on college students' AI literacy (Shen and Cui, 2024; Wang K. et al., 2025). Satisfying these media-related expectations readily awakens high-quality functional motivation, prompting individuals to invest more time and energy in technological interactions and gain more from them (Li et al., 2025), facilitating a deep translation of AI literacy at the cognitive and skill levels (Li et al., 2026).

Exploring the antecedent variables of such multidimensional motivation reveals that the normalized use of AI and the resulting positive learning perceptions show a significant positive correlation with students' motivation (Galindo-Domínguez et al., 2025). Concurrently, core value appraisal dimensions, including attainment value, perceived utility, intrinsic interest, and perceived cost, along with expectancy levels, collectively constitute key indicators predicting AI adoption motivation (Yurt and Kasarci, 2024). Essentially, these value beliefs relate to an individual's overall attitude tendency toward AI. Just as students holding positive attitudes toward distance education environments exhibit higher academic engagement (Koca et al., 2024), learners who maintain positive attitudes toward AI similarly unleash stronger curiosity and a sustained willingness to inquire (Sultana et al., 2025). An individual's positive attitude toward a specific activity strengthens their behavioral intentions. This attitude prompts them to actively seek the fulfillment of instrumental, entertainment, social, and escape gratifications in practice. When learners hold open and trusting beliefs about AI, they are more likely to establish a sense of autonomous control in human-machine collaborative contexts and build competence through dynamic interactive feedback (Chiu et al., 2024). Recent research also indicates that a positive attitude, viewing AI as an empowering tool rather than an external threat, can significantly benefit learners' sense of psychological control over AI use, opportunities for active choice in AI use, and entertainment gratification in AI use (Liang and Reiss, 2025). Users with such positive attitudes tend to view technological interactions as opportunities to expand cognitive boundaries rather than as mere learning burdens. This cognitive identification and emotional resonance can effectively buffer the psychological pressure brought by external performance goals. Consequently, learners become willing to exert more cognitive effort for deep understanding, transforming the coercive sense of external discipline into structured utilization motives within autonomous learning contexts (Jin et al., 2025). Therefore, a positive AI attitude can effectively awaken learners' motivations by fostering clear outcome expectations. This goal-directed drive then serves as a critical mediating hub, prompting individuals to engage in deep technological participation behaviors and ultimately achieve a substantial enhancement in AI literacy.

### AI self-efficacy and AI motivation as potential sequential mediators

Existing empirical research widely confirms that general self-efficacy not only positively predicts learning motivation but also fosters persistent goal-directed drive by reinforcing the individual's experience of accomplishment (Jia and Tu, 2024). Learners with high self-efficacy often demonstrate outstanding academic performance, largely attributable to the close connection between efficacy beliefs and learning motivation (Tang and He, 2023). An individual's exploratory drive across instrumental and recreational domains is substantively awakened only when they are confident in their ability to control complex situations or master specific skills. When facing emerging tools like AI, which present a relatively high technological threshold and uncertainty, a sense of capability often serves as a prerequisite for the intention of continued use. Previous studies have confirmed that technology-dimension self-efficacy constitutes a key antecedent variable for learning motivation, where learners' positive pre-assessments of their technological mastery directly translate into a willingness for deep inquiry (Liwanag and Galicia, 2023). Research by Teng et al. (2023) further reveals that the core mechanism by which self-efficacy drives motivation lies in its profound reshaping and maintenance of students' learning interests. During the application of AI, the positive AI attitude formed by individuals based on task value appraisals first builds their initial efficacy expectations. This escalating AI self-efficacy precisely fills the gap in college students' expectancy requirements when confronting complex intelligent tools. The adequate establishment of confidence further prompts strong motivations for technological utilization. Driven by this continuous and purposeful engagement orientation, learners gradually transcend the superficial limitations of tool substitution, allocating more cognitive resources toward cognitive restructuring and thereby completing a substantive leap toward higher-order AI literacy. In summary, this study aims to explore a potential sequential mediating pathway, examining whether AI attitude benefits college students' AI self-efficacy, which in turn bolsters their AI learning motivation, ultimately improving their AI literacy.

### The present study

Although existing literature has preliminarily confirmed the positive promotional effects of independent psychological variables, including attitude, self-efficacy, and motivation, on AI literacy, current research is mostly confined to exploring fragmented associations among these elements. It has not yet fully elucidated the micro-level psychological trajectory from an individual's initial technological perception to the transformation of complex comprehensive capabilities. Faced with the unstructured interaction challenges brought by AI, college students undergo profound internal cognitive restructuring and motivational shifts during the process of technological adaptation. Relying solely on static binary relationship models clearly cannot holistically capture this dynamic and complex psychological transition. To address the research gaps in this field, the present study deeply integrates self-efficacy theory and Uses and Gratifications Theory to construct a multiple sequential mediation model. It aims to systematically examine how college students' AI attitude substantively influences their AI literacy level through the chain mechanism of AI self-efficacy and AI motivation. Through this research, we aim to overcome the limitations of previous studies that emphasized technology acceptance while neglecting psychological development, systematically revealing the complete chain from cognitive appraisal, efficacy beliefs, and intrinsic drive to capability generation. This study outlines the implicit mechanisms of learners' literacy development in the intelligent era, significantly expanding the explanatory boundaries of relevant psychological theories within complex educational technology scenarios. Accordingly, the following research hypotheses are proposed:

**H**_**1**_: AI attitude is directly and positively associated with AI literacy.

**H**_**2**_: AI attitude is indirectly and positively associated with AI literacy through the mediation of AI self-efficacy.

**H**_**3**_: AI attitude is indirectly and positively associated with AI literacy through the mediation of AI motivation.

**H**_**4**_: AI attitude is indirectly and positively associated with AI literacy through the serial mediations of AI self-efficacy and AI motivation.

The research model is shown in Figure 1.

**Figure 1:**
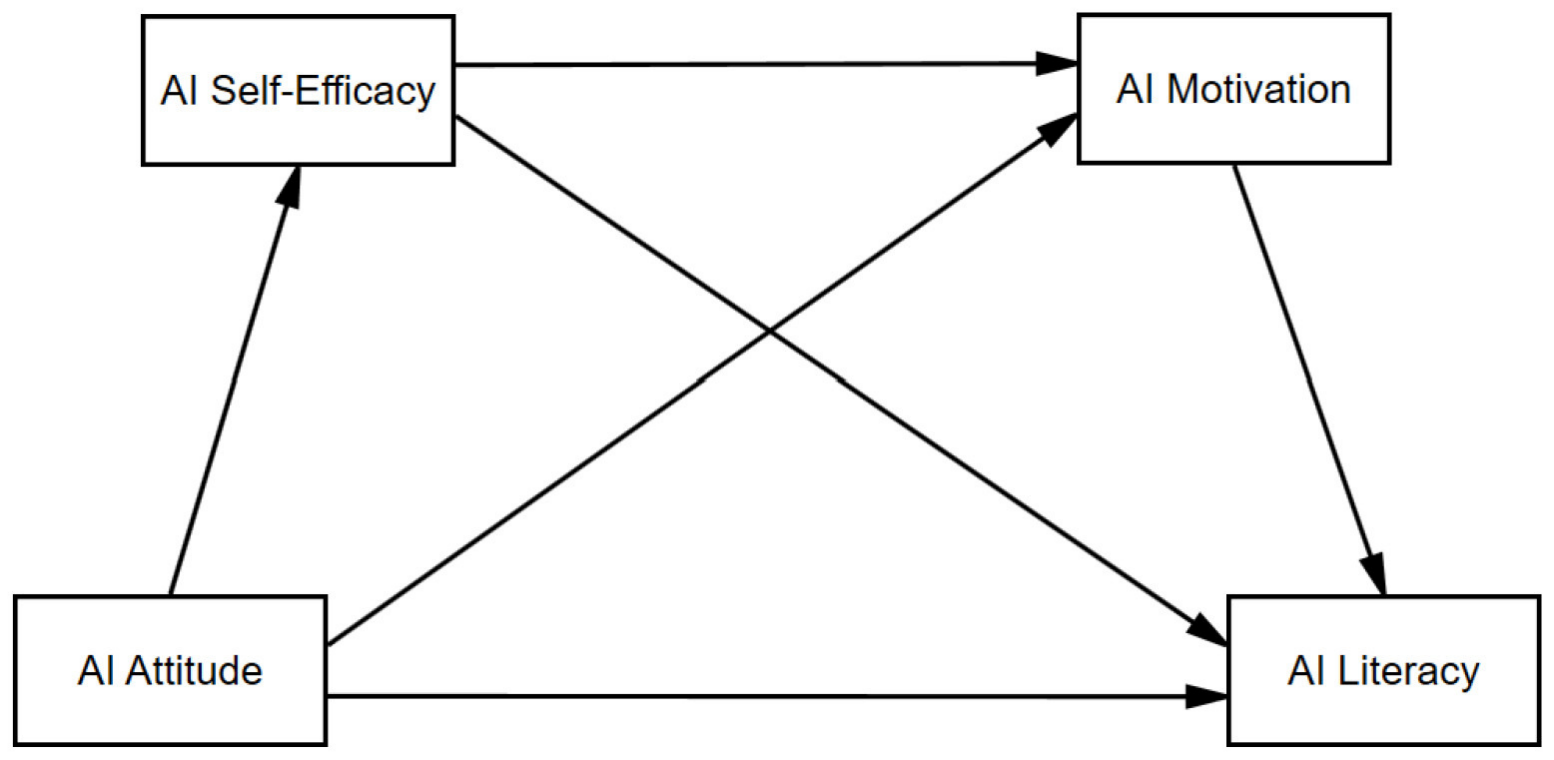
Research model.

## Methods

### Participants

The participants in this study were recruited from two universities in Shandong Province, China (one specializing in natural sciences, and the other in humanities and social sciences). A cluster sampling method was utilized to select the participants, specifically targeting freshmen, sophomores, juniors, and seniors of undergraduate students. Recruitment was conducted from December 2025 to January 2026, during the first semester of the 2025–2026 academic year. The survey was administered electronically via the online platform of SoJump (Wenjuanxing). The research team sent assistance requests to class counselors of potential participant classes. The participant composition included 10 classes of freshmen, 12 classes of sophomores, 5 classes of juniors, and 3 classes of seniors. All participants volunteered to take part in the study and were fully informed of the research purpose, data confidentiality measures, and their right to withdraw from the study at any time. The questionnaire completion time was approximately 8 to 10 min per participant.

A total of 1,453 undergraduate students expressed willingness to participate. Ultimately, 1,372 questionnaires were returned and initially considered valid, resulting in a response rate of 94.43%. Among these, 38 respondents were excluded employing listwise deletion, meaning that any participant with a missing value on any item was deleted from the analysis. A further 11 respondents were excluded because of inattentive or invariant responding. Cases with identical ratings across all items were not treated as statistical outliers. They were classified as inattentive or invariant responding because the instruments included positively and negatively worded items and measured multiple dimensions, so identical responses across all items indicated that respondents did not differentiate item content. These cases were excluded as a data quality control step. After these exclusions, the final sample size included in the data analysis was 1,323 respondents (*N* = 597 for males and *N* = 726 for females; *N* = 486 for freshmen, *N* = 535 for sophomores, *N* = 209 for juniors, and *N* = 93 for seniors). All participants were assured of anonymity and provided informed consent prior to participation.

### Measures

For instruments not originally developed in Chinese, we followed a translation and back translation procedure. Two bilingual researchers independently translated the items into Chinese. A third bilingual researcher, who was blind to the original items, back translated the Chinese version into English. An expert panel including two psychology faculty members and four bilingual graduate students compared the original and back translated versions and resolved discrepancies through discussion. Cultural adaptation focused on academic and campus situations familiar to Chinese university students and on wording that would be understood similarly across grade levels. A pilot test was conducted with 120 undergraduate students who were not included in the final sample. They completed the questionnaire and provided feedback on item clarity, comprehension, and response format. Based on these feedbacks, minor wording revisions were made to improve item clarity and ensure that the items were appropriately understood by the target respondents prior to the formal survey.

#### AI attitude

To evaluate college students' attitudes toward AI, this study utilized the AI Attitude Scale (AIAS-4) developed by Grassini (2023a). The AIAS-4 is a unidimensional instrument comprising four items (e.g., “I think AI technology is positive for humanity”). A 5-point Likert scale ranging from 1 (strongly disagree) to 5 (strongly agree) was used for scoring. Higher scores indicate a more positive attitude toward AI. In the current study, the Cronbach's α coefficient was 0.95.

#### AI self-efficacy

AI self-efficacy was assessed using the AI Self-Efficacy Scale developed by Carolus et al. (2023). This 6-item scale consists of two sub-dimensions: AI problem solving (e.g., “I can rely on my skills in difficult situations when using AI”) and AI learning (e.g., “I can keep up with the latest innovations in AI applications”). A 5-point Likert scale ranging from 1 (strongly disagree) to 5 (strongly agree) was used for scoring, with higher total scores representing greater levels of AI self-efficacy. The Cronbach's α coefficient in the present study was 0.96.

#### AI motivation

To measure the motivations behind AI usage, we employed the AI Use Motivation Scale designed by Huang et al. (2024). This instrument includes 12 items categorized into four sub-dimensions: AI escape motivation (e.g., “I use AI to escape stress and responsibility”), AI social motivation (e.g., “I use AI because I need to interact with someone”), AI instrumental motivation (e.g., “I use AI to enhance my learning and knowledge”), and AI entertainment motivation (e.g., “I use AI because it provides me with a variety of entertainment activities”). A 4-point Likert scale ranging from 1 (strongly disagree) to 4 (strongly agree) was used for scoring. Higher total scores reflect stronger motivation to use AI. In this study, the Cronbach's α coefficient for the scale was 0.91.

#### AI literacy

College students' AI literacy levels were evaluated using the AI Literacy Scale (AILS) formulated by Wang et al. (2023). This scale comprises 12 items distributed across four sub-dimensions: awareness (e.g., “I can distinguish between smart devices and non-smart devices”), usage (e.g., “I can skillfully use AI applications or products to help me with my daily work”), evaluation (e.g., “I can choose a proper solution from various solutions provided by a smart agent”), and ethics (e.g., “I always comply with ethical principles when using AI applications or products”). A 7-point Likert scale ranging from 1 (strongly disagree) to 7 (strongly agree) was used for scoring, with higher total scores indicating superior levels of AI literacy. The Cronbach's α coefficient in the current investigation was 0.87.

#### Covariates

In this study, gender and income were used as covariates. Gender was coded as 0 = girls, 1 = boys. Income was coded as 1 = less than 50,000 yuan each year; 2 = 50,000–100,000 yuan each year; 3 = 100,000–200,000 yuan each year; and 4 = more than 200,000 yuan each year. At the time of data collection, the approximate exchange rate was 1 USD = 7 CNY. Gender and income were included in all the analytical processes, covering descriptive statistics, correlational analysis, and the test of the serial mediation model.

### Analytical procedures

The data were analyzed by constructing the structural associations to explore the direct and indirect paths among AI attitude, AI self-efficacy, AI motivation, and AI literacy, with SPSS 25.0 software. The mediation effects were investigated using Model 6 from the Process 4.0 macro, specifying the variables as follows: X = AI attitude, M1 = AI self-efficacy, M2 = AI motivation, and Y = AI literacy. The bootstrapping bias-corrected confidence interval (CI) procedure was employed to examine mediation effects by utilizing 5,000 bootstrap samples (Hayes, 2013).

## Results

### Descriptive statistics and Pearson correlations

Table 1 showed the results of descriptive statistics and Pearson correlations for all study variables. The mean values of AI attitude, AI self-efficacy, AI motivation, and AI literacy were 3.939, 3.802, 2.525, 4.932, respectively. In terms of Pearson correlations, gender exhibited a significantly positive correlation with AI attitude, AI self-efficacy, AI motivation, and AI literacy, but an insignificant correlation with income; income displayed a significantly positive correlation with AI attitude, AI self-efficacy, and AI literacy, but an insignificant correlation with AI motivation; AI attitude was significantly and positively correlated with AI self-efficacy, AI motivation, and AI literacy; AI self-efficacy was significantly and positively correlated with AI motivation and AI literacy; as well, AI motivation was significantly and positively correlated with AI literacy. The correlation coefficients among all study variables ranged from 0.013 to 0.845, all below the recommended acceptable criterion of 0.850 (Kline, 2016). Also, we tested a variance inflation factor (VIF) for the current model. The results showed that the VIF values of all independent variables ranged from 1.130 to 3.549, well below the common threshold value of 10, further showing that there was no serious multicollinearity problem in the current model and thus the statistical results were reliable (Hayes, 2013; Kline, 2016).

**Table 1:** Descriptive statistics and Pearson correlations among all variables.

Variables	*M*	*SD*	1	2	3	4	5	6
1. Gender	0.450	0.498	_					
2. Income	1.920	0.933	0.052	_				
3. AI attitude	3.939	0.840	0.063^*^	0.077^**^	_			
4. AI self-efficacy	3.802	0.857	0.074^**^	0.080^**^	0.845^**^	_		
5. AI motivation	2.525	0.607	0.083^**^	0.013	0.323^**^	0.329^**^	_	
6. AI literacy	4.932	1.011	0.089^**^	0.074^**^	0.725^**^	0.780^**^	0.407^**^	_

^*^p < 0.05; ^**^p < 0.01. Gender and income are covariates. Gender was coded as 0 = girls, 1 = boys. Income was coded as 1 = less than 50,000 yuan each year; 2 = 50,000–100,000 yuan each year; 3 = 100,000–200,000 yuan each year; and 4 = more than 200,000 yuan each year. At the time of data collection, the approximate exchange rate was 1 USD = 7 CNY.

More than that, convergent validity and discriminant validity were examined to establish that the constructs were distinct from one another and that the items measured the same construct. Convergent validity was assessed using composite reliability (CR) and average variance extracted (AVE). As shown in Table 2, CR values for AI attitude, AI self-efficacy, AI motivation, and AI literacy ranged from 0.764 to 0.960, all exceeding the threshold of 0.60; meanwhile, AVE values for all variables ranged from 0.494 to 0.832, all surpassing the acceptable criterion of 0.36, thereby indicating good convergent validity for AI attitude, AI self-efficacy, AI motivation, and AI literacy (Lam, 2012; Niu, 2026; Zhang and Zheng, 2021). Additionally, Table 2 shows that the square root of the AVE for each construct was greater than the correlations between that construct and other constructs, indicating that the measurement models for AI attitude, AI self-efficacy, AI motivation, and AI literacy had satisfactory discriminant validity (Voorhees et al., 2016).

**Table 2:** Convergent and discriminant validity for all variables.

Constructs	Convergent validity		Discriminant validity			
	CR	AVE	AI attitude	AI self-efficacy	AI motivation	AI literacy
AI attitude	0.952	0.832	**0.912**			
AI self-efficacy	0.960	0.802	0.845^**^	**0.896**		
AI motivation	0.764	0.494	0.323^**^	0.329^**^	**0.703**	
AI literacy	0.898	0.688	0.725^**^	0.780^**^	0.407^**^	**0.829**

CR, composite reliability; AVE, average variance extracted. Bold diagonal values represent the square root of AVE. ^**^p < 0.01.

### Test of the chain mediation model

As seen from Table 3 and Figure 2, the results demonstrated that AI attitude is positively associated with AI self-efficacy (β = 0.860, *p* < 0.001), AI motivation (β = 0.114, *p* < 0.01), and AI literacy (β = 0.243, *p* < 0.001); AI self-efficacy is positively associated with AI motivation (β = 0.136, *p* < 0.001) and AI literacy (β = 0.655, *p* < 0.001); as well, AI motivation is positively correlated with AI literacy (β = 0.262, *p* < 0.001). Additionally, as seen from Table 4, the first indirect path (i.e., AI attitude → AI self-efficacy → AI literacy) displayed a significantly positive effect [β = 0.563, *SE* = 0.040; Bias-corrected CI (95%) = (0.485, 0.640)]. The second indirect path (i.e., AI attitude → AI motivation → AI literacy) showed a significantly positive effect [β = 0.030, *SE* = 0.011; Bias-corrected CI (95%) = (0.011, 0.051)]. Likewise, the third indirect path (i.e., AI attitude → AI self-efficacy → AI motivation → AI literacy) embodied a significantly positive effect [β = 0.031, *SE* = 0.011; Bias-corrected CI (95%) = (0.013, 0.054)]. Given that classroom identifiers were not retained and cluster sampling dependencies could not be modeled, these observed associations cannot be considered definitive and should be treated as preliminary factors requiring replication using hierarchically structured data with cluster identifiers.

**Table 3:** The unstandardized results of regression analysis among all variables.

Outcome variables	Explanatory variables	Path coefficients	*SE*	*T*	Fit index *R*	*R^2^*	*F*
AI self-efficacy	Gender	0.035	0.025	1.366	0.846	0.715	1,105.037
	Income	0.013	0.014	0.960			
	AI attitude	0.860^***^	0.015	57.122			
AI motivation	Gender	0.073^*^	0.032	2.295	0.345	0.119	44.502
	Income	−0.012	0.017	−0.684			
	AI attitude	0.114^**^	0.035	3.268			
	AI self-efficacy	0.136^***^	0.034	3.964			
AI literacy	Gender	0.043	0.034	1.297	0.804	0.647	482.194
	Income	0.012	0.018	0.650			
	AI attitude	0.243^***^	0.037	6.568			
	AI self-efficacy	0.655^***^	0.036	17.978			
	AI motivation	0.262^***^	0.029	9.028			

^*^p <0.05; ^**^p <0.01; ^***^p <0.001. Gender and income are covariates. Gender was coded as 0 = girls, 1 = boys. Income was coded as 1 = less than 50,000 yuan each year; 2 = 50,000–100,000 yuan each year; 3 = 100,000–200,000 yuan each year; and 4 = more than 200,000 yuan each year. At the time of data collection, the approximate exchange rate was 1 USD = 7 CNY.

**Figure 2:**
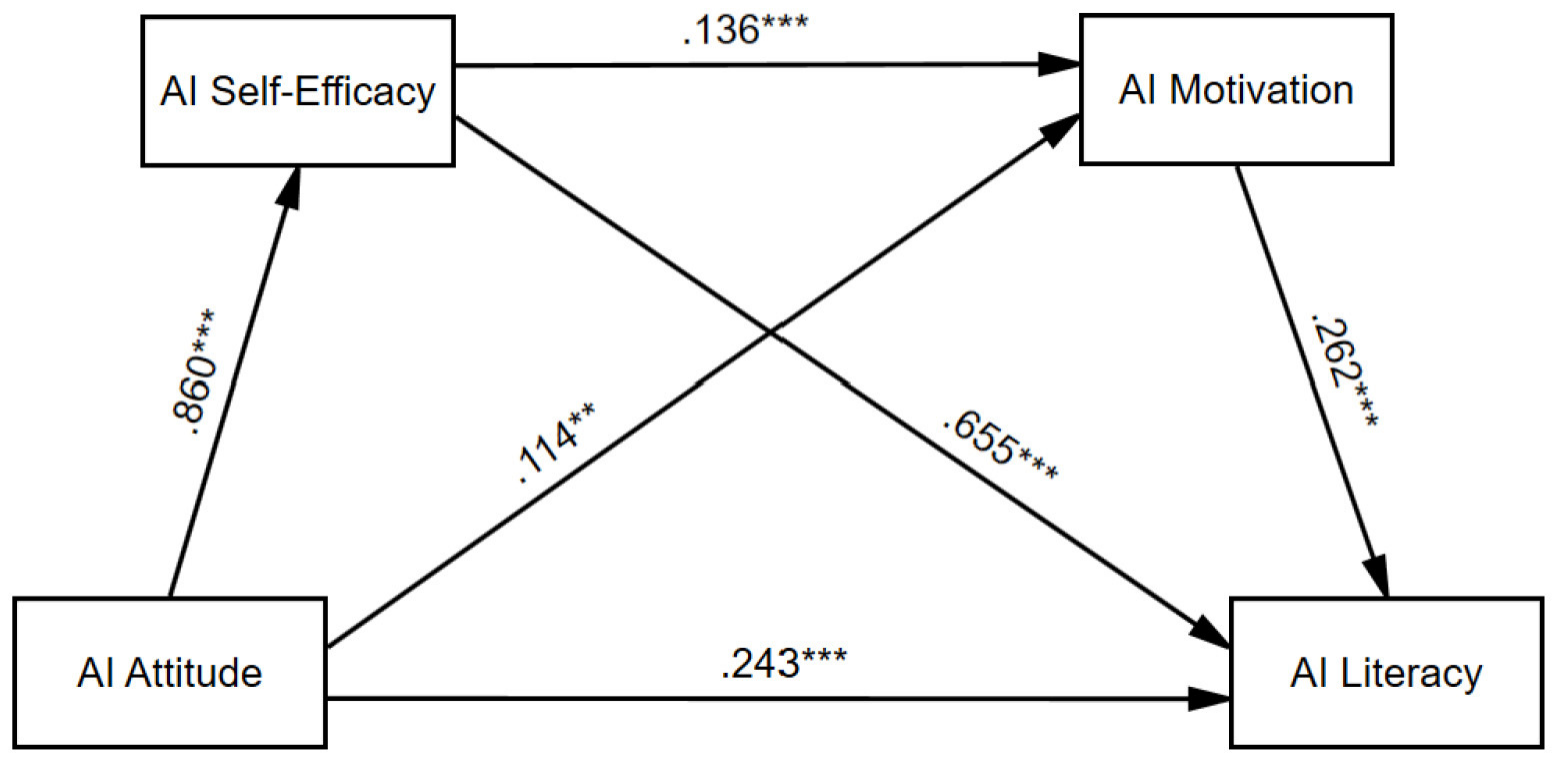
The estimated model. ^**^p < 0.01; ^***^p < 0.001.

**Table 4:** The unstandardized results of mediating effects among study variables.

Paths	β	*SE*	Bias-corrected CI (95%)	
			Lower	Upper
AI attitude → AI self-efficacy → AI literacy	0.563^***^	0.040	0.485	0.640
AI attitude → AI motivation → AI literacy	0.030^**^	0.011	0.011	0.051
AI attitude → AI self-efficacy → AI motivation → AI literacy	0.031^**^	0.011	0.013	0.054

^**^p <0.01; ^***^p <0.001.

## Discussion

The present study explored the structural relationships between AI attitude, AI self-efficacy, AI motivation, and AI literacy among contemporary college students. The findings revealed that AI attitude is positively associated with AI literacy, and this observed association was consistent with H_1_ under the assumption of the analysis. AI self-efficacy showed a positively indirect association between AI attitude and AI literacy, and this pattern was consistent with H_2_ under the assumption of the analysis. AI motivation showed a positively indirect association between AI attitude and AI literacy, and this pattern was consistent with H_3_ under the assumption of the analysis. AI self-efficacy and AI motivation showed a positively serial indirect association between AI attitude and AI literacy, and this pattern was consistent with H_4_ under the assumption of the analysis. All the above findings are preliminary associations that require replication using appropriately recorded and analyzed hierarchical data. These findings collectively highlight the critical function of AI attitude its association with AI literacy through both independent and sequential mediating pathways. Building on these results, by acknowledging that the measurement captures subjective cognitive appraisals and self-perceptions rather than objective technical performance, and taking into account the methodological constraints imposed by unmeasured classroom-level clustering, the following discussion interprets these associations and situates them within the broader landscape of existing research on internal psychological factors of AI competency development in the digital age.

The direct association indicated that AI attitude is positively related to AI literacy, reflected by some prior studies (e.g., Chang and Hwang, 2025; Hornberger et al., 2023; Mansoor et al., 2024; Park, 2026; Wang et al., 2023). Actually, the direct positive association between AI attitude and AI literacy reflects a theoretically grounded psychological mechanism that extends beyond simple bivariate correlation. According to recent systematic review evidence, AI literacy demonstrates robust positive correlations with positive AI attitudes across diverse populations and measurement contexts (Bewersdorff et al., 2025b). This relationship can be explained through multiple intertwined pathways. From a cognitive-affective perspective, positive AI attitudes may function as affective filters associated with reduced technology-related anxiety and psychological resistance (Schepman and Rodway, 2023), thereby freeing cognitive resources for deeper engagement with AI concepts rather than expending them on threat appraisal. When students perceive AI as beneficial rather than threatening, they are more willing to invest sustained effort in building subjective confidence regarding algorithmic principles, critically evaluating AI-generated outputs, and reflecting on ethical applications, all core dimensions of self-evaluated AI literacy (Kong et al., 2023; Long and Magerko, 2020). Furthermore, positive attitudes are associated with approach-oriented behavioral tendencies that facilitate exploratory learning, enabling individuals to accumulate experiential knowledge through active engagement with AI tools (Bewersdorff et al., 2025a). The reciprocal reinforcement between attitude and literacy operates through self-perpetuating feedback loops: Positive attitudes are associated with engagement, engagement is in turn related to favorable self-appraisals of competence, and these subjective gains are correlated with positive attitudes toward the technology (Ning et al., 2025). More than that, comparative empirical cases also support this conclusion. For example, a cross-national study covering India, Saudi Arabia, Malaysia, and Egypt showed that attitudes toward AI are positively connected to perceived AI literacy/competency (Mansoor et al., 2024). Clearly, these theoretical mechanisms and cross-national empirical findings underscore that AI attitude is not merely a peripheral disposition but rather a fundamental psychological factor associated with perceived AI literacy development. This highlights the necessity of intentionally cultivating positive attitudes as a strategic educational priority, positioning attitudinal interventions alongside skill-based instruction to foster comprehensive and enduring perceived AI competencies.

This study found that AI self-efficacy plays a significant mediating role between AI attitude and AI literacy. This result is consistent with a context in which affective evaluations and cognitive beliefs are associated with competency building at the mercy of intelligent technology. Rather than a superficial preference, a positive attitude may serve as an emotional foundation associated with reduced technological uncertainty and stronger cognitive control (Scherer et al., 2019). Such a favorable affective stance provides the sustained psychological energy needed for individuals to maintain confidence when facing complex technological challenges and is thereby associated with stronger self-efficacy (Sassis et al., 2021). This finding profoundly reflects the patterns of psychological adaptation among learners during technological shifts and aligns with recent research by Atalla et al. (2025). Furthermore, this conclusion has been cross contextually validated by studies on distance education for postgraduates in Australia (Prior et al., 2016) and computer applications among American college students (Torkzadeh et al., 2006). Both demonstrate that students with a favorable outlook toward new web technologies exhibit much greater gains in self-efficacy than those with negative attitudes. Specifically, this evaluation-related efficacy belief is associated with persistence when handling algorithmic tasks and encourages individuals to translate firm task beliefs into sustained effort and confident judgment during actual interactions (Wang and Chuang, 2024). Such behavioral engagement is directly related to the development of self-rated literacy. These results corroborate the work of Kong et al. (2025) on the drivers of perceived AI literacy and confirm the pivotal predictive role of self-efficacy in competency acquisition models. Empirical evidence from diverse settings including e health literacy among nursing students in South Korea (Jeon and Kim, 2022) and information literacy among university students from European countries including Denmark, Finland, France, Germany, Italy, Luxembourg, Portugal, and Russia (Mirazchiyski, 2025) consistently shows that efficacy beliefs are connected to literacy development. Taken together, these examples suggest that the psychological acceptance of emerging technologies is linked to subjective evaluation outcomes through the reinforcing bridge of self-efficacy. This highlights how psychological empowerment is associated with the formation of capability perceptions.

The present study also identified an independent mediating path of AI motivation between AI attitude and AI literacy and described how affective evaluations are associated with the acquisition of high-level skills by activating expectations that AI use can satisfy instrumental, entertainment, social, and escape motives. When individuals hold highly positive appraisals, these cognitive assessments are correlated to stronger expectations of gratifying AI use and a greater readiness to employ AI for task completion, enjoyment, social connection, and affect management (Sultana et al., 2025) to form the basis for motivation. This aligns with the findings of Bernaus and Gardner (2008) in second language acquisition and suggests that whether in traditional classrooms or intelligent environments, a high value assessment of the target object is a prerequisite for intentional engagement. Surveys on AI tool usage among Turkish students (Yurt and Kasarci, 2024) and AI learning tasks among UK students (Liang and Reiss, 2025) further support the association between positive attitudes and motivational arousal. Further analysis reveals that such evaluation driven motivation is associated with deeper psychological investment when processing complex information and is associated with greater cognitive flexibility and persistence (Li et al., 2025). This depth is crucial for constructing self-evaluated literacy. Findings among specific groups such as medical students also suggest that the strength of motivation is associated with the allocation of cognitive resources during the competency building process (Li et al., 2026). Additionally, research on Information and Communication Technology (ICT) literacy in Germany (Senkbeil and Ihme, 2017) and digital literacy in Malaysia (Lilian, 2022) indicates that motivational levels are related to the depth of literacy acquisition. These cross-cultural empirical studies collectively show that positive affective attitudes are linked to substantive self-assessed literacy by activating the will to act.

By constructing a chain mediation model, this study explored a pattern of associations from attitude to literacy where the relationship between AI attitude and AI literacy is associated with sequential indirect paths through AI self-efficacy and AI motivation. This path is consistent with a hypothesized sequence in which students' initial affective evaluation may precede a belief in their own competence which may then relate to a sustained desire to explore and may ultimately be associated with subjective competency building. The cross-sectional design cannot establish temporal precedence or causal direction. This finding aligns closely with the core tenets of self-efficacy theory and Uses and Gratifications Theory. According to self-efficacy theory (Bandura, 1997), evaluative attitudes are theorized to precede cognitive beliefs. A positive appraisal may act as a proactive regulator that is associated with reduced perceived threat of algorithmic logic and provides the cognitive support necessary to establish self-efficacy (Zimmerman, 2000; Wang and Chuang, 2024). This is consistent with earlier discussions on internet attitudes (Wu and Tsai, 2006) and efficacy belief mechanisms (Ng et al., 2024) and explores the theoretical pattern of evaluations being associated with beliefs. Building on this and guided by U&G (Katz et al., 1973), this study finds that the establishment of self-efficacy is related to learners to proactively seek specific fulfillments from AI. Users who are confident in their technological capabilities tend to develop diverse AI-use motivations, encompassing instrumental, entertainment, social, and escape needs (Smock et al., 2011). This multi-dimensional motivational factor is linked to long-term technological exploration. Because emerging AI systems involve inherent complexity, a strong sense of self-efficacy is associated with reduced psychological avoidance and maintains continuous learning engagement (Teng et al., 2023; Shi and Zhang, 2025). Driven by the pursuit of these various gratifications, students gradually progress past basic technical operations. They begin to allocate higher-order cognitive resources toward critical analysis and ethical reflection, and are associated with overall perceived AI literacy (Kong et al., 2023; Su et al., 2023).

Beyond statistical significance, an evaluation of the association magnitudes reveals an empirical hierarchy among these psychological mechanisms. As detailed in the mediation analysis, the indirect association operating solely through AI self-efficacy is substantially larger than both the independent motivational pathway and the serial motivational pathway. This difference in magnitude warrants explicit discussion. Motivation often serves as an initial catalyst for technological engagement. However, the enhancement of self-evaluated AI literacy involves overcoming steep learning curves, mastering algorithmic logic, and applying complex problem-solving skills. According to Bandura (1997), when individuals confront highly complex cognitive tasks, robust capability beliefs are significantly more predictive of sustained effort and subjective proficiency evaluation than the mere presence of behavioral motives. Recent research in digital education further corroborates this phenomenon, demonstrating that specific technological self-efficacy is associated with favorable self-perceptions of literacy stronger than general use motivations (Scherer et al., 2019). Therefore, having the concrete confidence to overcome algorithmic hurdles is associated with the sustained cognitive persistence required for higher self-evaluations of literacy. The notably smaller association sizes for the motivational pathways do not diminish their theoretical relevance, as they still accurately capture the essential gratifications sought by users. It is crucial to note that the magnitude of this independent motivational pathway (β = 0.030) is relatively modest. This suggests that while motivation is a necessary theoretical component for initiating action, the smooth progression from attitude to motivation and then to heightened self-assessed capability may face certain psychological bottlenecks if not accompanied by a strong belief in one's technological capabilities. In summary, this sequential path clarifies the psychological associations from AI attitude to AI literacy. It suggests that fostering perceived AI literacy in the modern era should integrate attitude guidance, confidence building, and motivational arousal. Yet, educational interventions must place a prioritized emphasis on solidifying students' self-efficacy rather than relying solely on the arousal of interest or top-down technical instruction.

Several limitations should be acknowledged in this study. Firstly, because all variables, including AI attitude, AI self-efficacy, AI motivation, and AI literacy, were assessed through self-report scales, the findings reflect participants' subjective perceptions and cognitive evaluations rather than objective, performance-based demonstrations of AI competence, which may introduce potential social desirability bias and common method variance. Future studies could enhance validity by incorporating multiple data sources, such as teacher evaluations, peer assessments, parental observations, or objective indicators like digital activity logs. Secondly, the sole use of a quantitative approach, though useful for examining statistical relationships, may not fully capture the nuanced ways students experience and internalize AI-related learning process. Incorporating qualitative methods, including in-depth interviews, focus groups, or classroom observations, would provide richer insight into how AI attitude relates to perceived literacy development. Thirdly, the cross-sectional design restricts causal interpretations. Longitudinal research is therefore necessary to investigate temporal sequences and the dynamic interplay among AI attitude, AI self-efficacy, AI motivation, and AI literacy over time, thereby offering stronger evidence for potential causality. Finally, considering classroom identifiers were not available and classroom level dependence could not be modeled in the PROCESS analyses, all reported findings should be interpreted as exploratory associations requiring future replication using appropriately recorded and analyzed hierarchical data.

## Implications

### Theoretical implications

This study makes several theoretical contributions to the extant literature on AI literacy. Firstly, by integrating Bandura's self-efficacy theory and Katz et al.'s UGT, we proposed and tested a sequential mediation model that describes the nuanced psychological pathways through which AI attitude is related to perceived AI literacy. The analysis provides observed indirect associations consistent with this model rather than empirical validation of a temporal or causal process. This integrative framework moves beyond the fragmented, variable-centric approaches that have characterized prior research, which typically examined isolated bivariate relationships or single-mediator models (Asio, 2025; Li et al., 2025). By observing that AI self-efficacy and AI motivation are associated with independent and sequential indirect paths, our findings are consistent with a hypothesized chain of psychological associations. Positive AI attitudes are associated with confidence in handling AI-related tasks (self-efficacy), which is in turn associated with individuals' media-related expectations, and these expectations are linked to multidimensional utilization motives that relate to subjective literacy acquisition. This pattern describes statistical indirect associations, not a demonstrated temporal or causal sequence. This serial mediation mechanism, where efficacy is associated with the primary indirect path, offers a more ecologically valid representation of how internal psychological dynamics may function, addressing a critical gap in the literature that has largely neglected the sequential interdependencies and varying magnitudes among these constructs.

Secondly, this study contributes to the ongoing scholarly discourse by shifting the analytical lens from macro-level external interventions—such as family regulation, classroom climate, and instructional practices (Shen and Cui, 2024; Wang X. et al., 2025; Zhang et al., 2026)—toward a deeper understanding of endogenous micro-level psychological processes. Our findings underscore that the efficacy of external support structures ultimately depends on their capacity to activate and align with learners' internal psychological resources. This insight extends prior theoretical frameworks that have predominantly relied on technology acceptance models (Chen et al., 2020; Kamruzzaman et al., 2023; Ouyang et al., 2022), which emphasize the physical attributes and functional utilities of AI tools while overlooking the complex cognitive and affective transformations that learners undergo. By foregrounding the subjective psychological dimensions of AI engagement, namely attitude, self-efficacy, and motivation, this study advances a more holistic theoretical perspective that recognizes the development of self-assessed AI literacy as a deeply internalized and psychologically mediated process. The present analysis offers preliminary associations consistent with this perspective.

### Practical implications

The findings of this study yield actionable insights for educational practitioners, curriculum designers, and policymakers seeking to cultivate AI literacy among university students. By illuminating the sequential psychological pathways through which AI attitudes translate into perceived AI literacy via the independent and serial mediations of AI self-efficacy and AI motivation, the current model can provide a conceptual blueprint for future educational design. For educators, these insights offer guidance on structuring classroom experiences that deliberately foster confidence and intrinsic engagement; for curriculum developers, they underscore the importance of integrating attitudinal and motivational components alongside technical skill instruction; and for institutional policymakers, they highlight the necessity of creating supportive learning environments that nurture students' internal psychological resources. Collectively, these implications shift the focus from solely external instructional inputs toward a more holistic approach that recognizes self-rated AI literacy as fundamentally shaped by learners' subjective psychological experiences.

Firstly, given the critical role of AI attitude as the initiating gateway in the psychological chain, educational interventions should prioritize the development of positive and balanced attitudes toward AI before introducing technical skills training. This suggests that introductory AI courses should incorporate structured opportunities for students to explore the societal implications, ethical considerations, and practical benefits of AI in personally meaningful contexts. Educators might design reflection-based activities, such as guided discussions on AI's transformative potential in students' future careers or facilitated dialogues addressing common fears about job displacement and algorithmic bias. Such attitudinal interventions can help students construct realistic, affirmative perceptions that reduce psychological resistance and establish a receptive foundation for subsequent competency perception development. Importantly, these efforts should extend beyond one-time orientation sessions to include ongoing engagement with AI applications across the curriculum, allowing attitudes to be continuously shaped through authentic experiential learning.

Secondly, the mediation pathways identified in this study highlight the strategic importance of designing learning environments that deliberately foster AI self-efficacy. Because self-efficacy operates through mastery experiences, vicarious observation, and emotional arousal (Bandura, 1997), educational programs should scaffold students' AI learning experiences to ensure early and repeated success. This can be achieved through carefully sequenced tasks that progress from guided, low-stakes AI applications to increasingly complex, open-ended challenges. For instance, instructors might begin with structured tutorials on using AI tools for basic information retrieval or writing assistance, gradually advancing to projects requiring critical evaluation of AI-generated outputs and ethical reasoning about AI use in professional contexts. Additionally, peer learning arrangements that showcase successful AI applications by students with similar backgrounds can provide powerful vicarious experiences that bolster self-efficacy. Institutions might also consider establishing self-assessment mechanisms that allow students to track their growing competencies, thereby reinforcing efficacy beliefs through tangible evidence of progress.

Thirdly, the serial mediation results describe a statistically indirect association in which AI use motivation that encompasses instrumental, entertainment, social, and escape motives links efficacy beliefs to perceived literacy development within the mediation model. The present analysis estimated serial mediation and did not test moderation. The relative magnitude of the indirect effects does not establish that AI self-efficacy moderates the association between AI motivation and AI literacy, nor does it demonstrate that one educational intervention sequence is more effective than another. The results therefore suggest that confidence-related beliefs and motivational processes may be jointly relevant, but this possibility requires experimental or longitudinal testing. From the perspective of UGT, educators should not treat motivation as an independent training objective. Educational designs may consider supporting AI self-efficacy through task decomposition, mastery experiences, and constructive feedback, and may also seek to activate multidimensional use gratifications through AI tasks with independent choice, collaborative inquiry activities, and goal-oriented feedback. Superficial engagement driven solely by grade constraints cannot support the deep cognitive investment required for higher-order self-evaluated AI literacy. Integrating AI learning with real-world problems, career aspirations and disciplinary demands can continuously activate the four categories of use motives, and provide stable support for students' long-term exploration and critical reflection.

Fourthly, the comprehensive nature of AI literacy—encompassing awareness, usage, evaluation, and ethics (Wang et al., 2023)—calls for instructional approaches that integrate cognitive, affective, and behavioral dimensions simultaneously. Given our finding that AI self-efficacy is associated with a substantially stronger indirect association than motivational pathways, interventions may consider a self-efficacy informed strategy. This involves supporting capability beliefs while examining whether motivation is associated with high-level subjective literacy outcomes. For example, a semester-long course might begin with reflective sessions cultivating positive attitudes toward AI, followed by scaffolded technical skill-building activities designed to establish self-efficacy, then incorporate project-based learning that leverages motivation to tackle authentic problems requiring evaluative and ethical reasoning. Assessment strategies should similarly align with this developmental sequence, using formative feedback to support efficacy development and self-assessment opportunities to reinforce motivation. Finally, institutional policies and support structures—such as accessible AI learning resources, faculty development programs, and recognition mechanisms for perceived AI literacy achievements—can create an enabling environment that may support the positive psychological associations identified in this study. By attending to the interconnected psychological pathways from attitude to self-efficacy to motives and ultimately to subjective literacy, educational stakeholders can design more coherent, impactful interventions that equip university students with the AI competencies essential for academic success and lifelong learning in an increasingly intelligent world.

In summary, this study provides a dual academic contribution by advancing a holistic theoretical framework that unpacks endogenous psychological mechanisms through sequential mediation and by offering actionable pedagogical pathways for digital capability development. Theoretically, it shifts the scholarly focus from macro level external interventions to micro level cognitive and affective dynamics, highlighting how AI attitude, AI self-efficacy, and AI motivation interconnect. Practically, it outlines how AI attitudinal foundations, AI self-efficacy scaffolds, and AI motivational contributors can be systematically orchestrated to support university students' AI literacy. Nevertheless, it is essential to emphasize that all proposed educational interventions and curricular designs should be treated as theoretically informed hypotheses for future experimental evaluation, rather than as empirically validated instructional strategies.

## Conclusion

This study investigated the structural relationships between AI attitude, AI self-efficacy, AI motivation, and AI literacy among contemporary university students, revealing that AI attitude not only directly associates with AI literacy but also indirectly associates with it through the independent mediations of AI self-efficacy and AI motivation, as well as their sequential mediations. These findings are overall consistent with the view that the development of AI literacy can be framed as a subjective, psychologically grounded process in which attitudinal appraisals, efficacy beliefs, and motivational factors are positively associated with AI-related competency perceptions. By describing this sequential pattern of associations, the study challenges the prevailing emphasis on external instructional inputs and calls attention to the often-overlooked internal resources that learners bring to their engagement with AI technologies. In an era increasingly defined by human-machine collaboration, cultivating the psychological foundation of AI literacy demands more than curricular innovation; it requires a pedagogical commitment to nurturing learners' confidence, motivation, and critical attitudes alongside their technical proficiencies. Ultimately, this research invites educators and policymakers to reconsider AI education as a holistic endeavor that recognizes the learner as an active psychological agent whose attitudes, beliefs, and motivations show promising exploratory associations with favorable self-assessed competency.

## Data Availability

The raw data supporting the conclusions of this article will be made available by the authors, without undue reservation.
